# Taking a step down on the reconstruction ladder for head and neck reconstruction during the COVID-19 pandemic

**DOI:** 10.1186/s12893-021-01134-1

**Published:** 2021-03-08

**Authors:** Haroon Ur Rashid, Mamoon Rashid, Nasir Khan, Shayan Shahid Ansari, Noshi Bibi

**Affiliations:** 1 Plastic Surgery Department, Shifa International Hospital, Pitras Bukhari Road H-8, Islamabad, Pakistan; 2 Head and Neck, Shifa International Hospital, Islamabad, Pakistan

**Keywords:** Reconstruction in COVID 19 pandemic, Pedicled flaps in free flap era, Head and neck reconstruction

## Abstract

**Background:**

Most of the head and neck cancers are time-critical and need urgent surgical treatment. Our unit is one of the departments in the region, at the forefront in treating head and neck cancers in Pakistan. We have continued treating these patients in the COVID-19 pandemic with certain modified protocols. The objective of this study is to share our experience and approach towards head and neck reconstruction during the COVID-19 pandemic.

**Results:**

There were a total of 31 patients, 20 (64.5%) were males and 11 (35.4%) patients were females. The mean age of patients was 52 years. Patients presented with different pathologies, i.e. Squamous cell carcinoma n = 26 (83.8%), mucoepidermoid carcinoma n = 2 (6.4%), adenoid cystic carcinoma n = 2 (6.4%) and mucormycosis n = 1 (3%). The reconstruction was done with loco-regional flaps like temporalis muscle flap n = 12 (38.7%), Pectoralis major myocutaneous flap n = 8 (25.8%), supraclavicular artery flap n = 10 (32.2%) and combination of fore-head, temporalis major and cheek rotation flaps n = 1 (3%). Defects involved different regions like maxilla n = 11 (35.4%), buccal mucosa n = 6 (19.3%), tongue with floor of mouth n = 6 (19.3%), mandible n = 4 (12.9%), parotid gland, mastoid n = 3 (9.6%) and combination of defects n = 1 (3%). Metal reconstruction plate was used in 3 (9.6%) patients with mandibular defects. All flaps survived, with the maximum follow-up of 8 months and minimum follow-up of 6 months.

**Conclusion:**

Pedicled flaps are proving as the workhorse for head and neck reconstruction in unique global health crisis. Vigilant use of proper PPE and adherence to the ethical principles proves to be the only shield that will benefit patients, HCW and health system.

## Introduction

Corona virus (COVID-19) was first reported in December 2019 in the city of Wuhan, China, and soon it was declared as a global pandemic by the World Health Organization in March 2020 [1]. At the end of March 2020, the COVID-19 outbreak was announced in Pakistan, all the elective procedures were discouraged to conserve the resources and strategically contain the COVID-19 spread following guidelines from centers for disease control and prevention (CDC) [2]. All the other urgent and emergency surgical procedures are modified to overcome the burden on the health system.

In our department, surgeries were limited to the treatment of trauma and aggressive cancers. The risks and benefits for the patient were thoroughly evaluated, keeping in mind the safety of health care workers involved in the peri-operative care.

The majority of patients presented to us with advanced cancers, being referred from peripheral hospitals. Such patients could not be further delayed because holding-up treatment would have deleterious impact on functional and aesthetic outcomes. These patients fell in tier 3a and 3b category (high-acuity surgery for malignancies of the upper aero-digestive tract, cutaneous melanoma and high-risk cutaneous squamous cell carcinoma with non-delayable reconstructions for most defects involving the upper aerodigestive tract) according to Centers for Medicare and Medicaid services (CMS) surgical guide lines [3].

Our team has been playing a leading role in head and neck reconstruction for the last 20 years in Pakistan. Micro-vascular free tissue transfer is considered standard of care in head and neck reconstruction [4].

Hence, micro-vascular free tissue transfer is often our first choice for head and neck reconstruction. Since the COVID-19 pandemic started with its unique challenging crisis, we have to modify our practice by taking a step down on the reconstructive ladder. In this paper, we are presenting our experience of head and neck reconstruction with loco-regional pedicled flaps, during the COVID-19 pandemic.

## Material and methods

In a 3 months period from April 1st 2020 till July 1st 2020, 31 patients underwent reconstruction for post ablative head and neck defects who were included in the study. Loco-regional flaps were used in all patients. Two surgeons (head and neck surgeon and reconstructive plastic surgeon) and two assistants were involved in all cases. All patients were pre-operatively screened with PCR COVID-19 nasal swabs and high-resolution CT scan (HRCT) Chest. Only COVID-19 negative (screened with PCR and HRCT) patients were operated. Patients, in which these investigations were not done, like in emergency and life threatening situations, were considered COVID-19 positive and excluded from study. During surgery all the operating room staff was directed to use particulate respirator mask (e.g. N95), eye protective goggles/face shields, surgical disposable gowns and gloves. Duration of surgical exposure, outcomes in terms of flap loss, wounds' infection/dehiscence, and functional recovery were noted. In these procedures the involved staff COVID-19 status was checked with the PCR every 2 weeks. This is a retrospective study approved from the ethical committee (Reference: IRB# 300-1120-2020). An informed consent was taken from all the patients whose pictures were used for publication purposes.

### Quality of life questionnaire (QOL Q)

In this study European Organization for Research and Treatment of Cancer Quality of Life Questionnaire-Core 30 (EORTC QLQ-C30) version 3 (the validated Taiwan Chinese version) was employed. [5, 6] Out of 31 patients only 8 patients took part in answering the questionnaire due to COVID-19 restrictions. Patients completed the EORTC QLQ-C30 after the surgery at 6 months follow-up. The scores of QLQ-C30 items were linearly transformed to 0–100 scales. All scales were calculated according to EORTC scoring manual. [7, 8] Higher scores on functioning scales represent better functional outcome and high scores for symptom scales correspond to higher problems or symptoms.

## Results

There were a total of 31 patients, 20 (64.5%) were males and 11 (35.4%) patients were females. The mean age of patients was 52 years. Patients presented with different pathologies, i.e. squamous cell carcinoma n = 26 (83.8%), mucoepidermoid carcinoma n = 2 (6.4%), adenoid cystic carcinoma n = 2 (6.4%) and mucormycosis n = 1 (3%). The reconstruction was done with loco-regional flaps like temporalis muscle flap n = 12 (38.7%), pectoralis major myocutaneous flap n = 8 (25.8%), supraclavicular artery flap n = 10 (32.2%) and combination of fore-head, temporalis major and cheek rotation flaps n = 1 (3%). Defects involved different regions like maxilla n = 11 (35.4%), buccal mucosa n = 6 (19.3%), tongue with floor of mouth n = 6 (19.3%), mandible n = 4 (12.9%), parotid gland, mastoid n = 3 (9.6%) and combination of defects n = 1 (3%). Metal reconstruction plate was used in 3 (9.6%) patients with mandibular defects. Patients evaluated clinically at follow-ups. All flaps survived, with the maximum follow-up of 8 months and minimum follow-up of 6 months. At 1 month follow-up, good mucosalization was seen in intraoral flaps. Minor wound dehiscence was seen in 4 (12.9%) patients who were conservatively managed. Mean operating time was 207 min with a range of 140 min to 347 min, (mean resection time 106 min, reconstruction time 101 min). Mean hospital stay was 3 days. Tracheostomy was done in 17 (54.8%) patients, which were removed on the 3rd post-operative day. None of the HCW involved in these cases was infected with COVID-19. Long term data is not available due to short follow-up course. Table 1 shows patients characteristics of our study.

**Table 1:** Patients characteristics in this study

1	*Gender*	Total numbers	%	Mean age 52 years	
	Males	n = 20	64.5		
	Females	n = 11	35.4		
2	*Pathological indications*			Mean resection time	
	Squamous Cell Ca	n = 26	83.8	106 min	
	Adenocystic Ca	n = 2	6.4		
	Mucoepidermoid Ca	n = 2	6.4		
	Mucormycosis	n = 1	3		
3	*Types of flaps*			Mean reconstruction time	
	Temporalis muscle flap	n = 12	38.7	101 min	
	Pectoralis major muscle flap	n = 8	25.8		
	Supraclavicular artery flap	n = 10	32.2		
	Combinations of local flaps	n = 1	3		
4	*Defects site*			Complications	
	Maxilla (palate)	n = 11	35.4	Wound dehiscence	n = 4 (12.9%)
	Buccal mucosa	n = 6	19.3	Flap failure	n = 0 (0%)
	Tongue/ floor of mouth	n = 6	19.3	COVID Pneumonia	n = 0 (0%)
	Mandible	n = 4	12.9		
	Parotid and Mastoid region	n = 3	9.6		
	Combined defects Maxilla, nose and cheek	n = 1	3		

### EORTC QLQ-C30

Quality of life assessment using EORTC QLQ-C 30 in 8 patients is mentioned in Table 2. The mean global health status was 66.46. The functional scales values were high in social functioning, role functioning and emotional functioning representing in improvement of their overall function after treatment. The symptoms scales had low scores in most aspects except for pain (mean score 19.68), fatigue (mean score 24.39) and financial difficulties (mean score 45.75).

**Table 2:** Results of the EORTC QLQ-C30 version 3.0 for head and neck cancer survivors

	(n = 8) Mean values
*Functional scales*	
Physical functioning	74.14
Role functioning	86.45
Emotional functioning	80.63
Cognitive functioning	78.76
Social functioning	86.35
*Symptom scales/items*	
Fatigue	24.39
Nausea and vomiting	4.51
Pain	19.68
Dyspnea	8.21
Insomnia	2.34
Appetite loss	11.53
Constipation	14.12
Diarrhea	6.25
Financial difficulties	45.75
Global health status/QOL	66.46

Following are our cases (Fig. 1. Case 1, Fig. 2. Case 2, Fig. 3. Case 3, Fig. 4. Case 4, Fig. 5. Case 5, Fig. 6. Case 6, Fig. 7. Case 7) showing the pre-operative pictures, per-operative pictures and their immediate follow-ups. Figure 8 shows patients after rehabilitation period of 6 months.

**Fig. 1:**
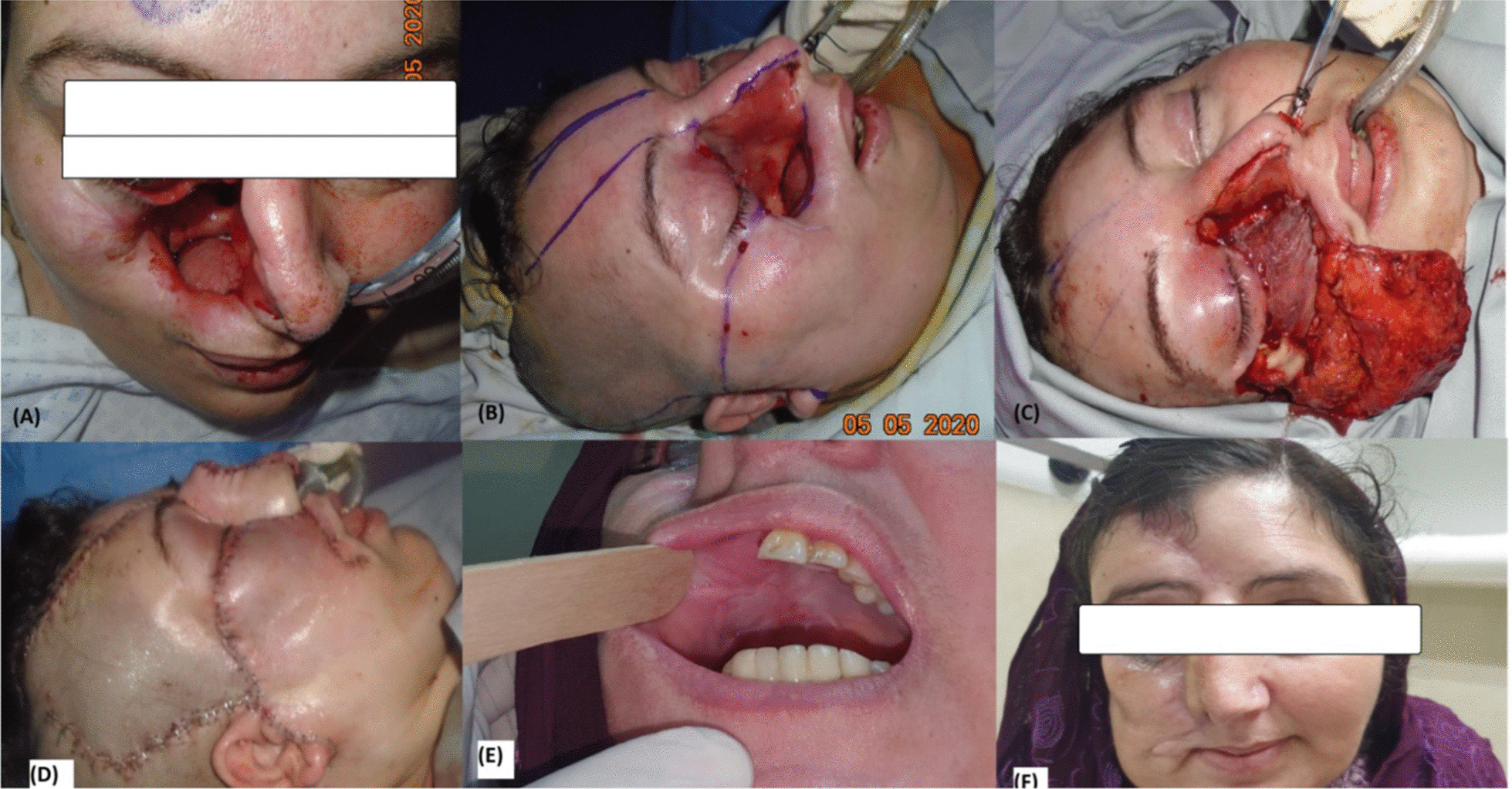
Case 1 **a** Young lady with large defect right cheek, nasal dorsum and palate with visible tongue at base. **b** Markings for forehead flap, temporalis muscle flap and cheek rotation advancement flaps. **c** Temporalis muscle flap inset done to reconstruct palate and fill the dead space. **d** Per-operative pic showing inset of forehead and cheek flaps. **e** Intra-oral view showing good mucosalization of temporalis muscle. **f** Frontal view of face at early follow up period

**Fig. 2:**
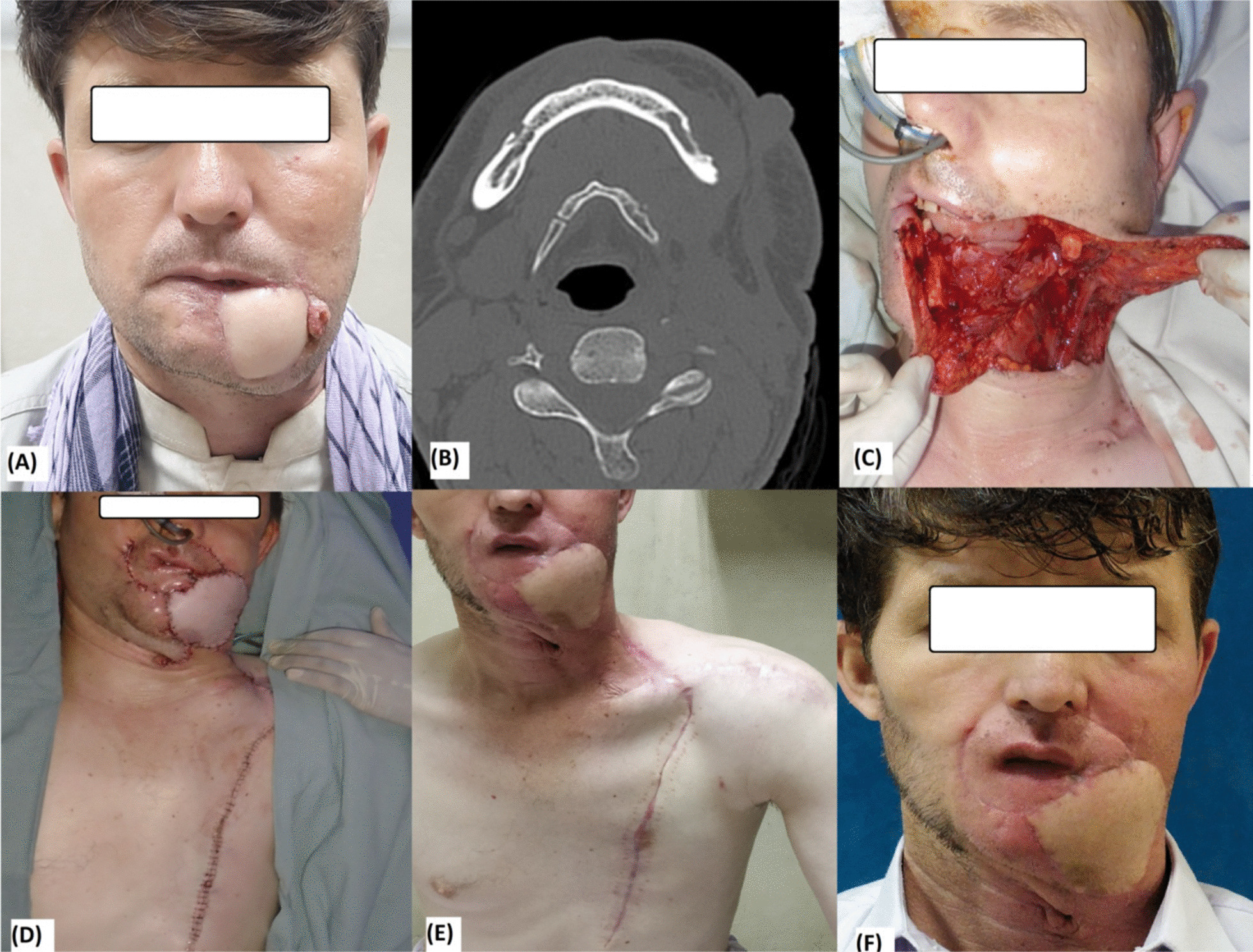
Case 2 **a** Case 2 Young male with recurrent Squamous cell carcinoma lower lip and mandible. There is a visible supraclavicular flap of previous surgery. **b** CT scan showing extent of tumor to bone and floor of mouth. **c** Per-operative view of wide local excision showing soft tissue and boney defect. **d** Per-operative view showing pectoralis major myocutaneous flap after inset and Karapandzic technique used for lip defect. **e** Early followup picture. **f** Frontal view of patient showing nicely healed wounds

**Fig. 3:**
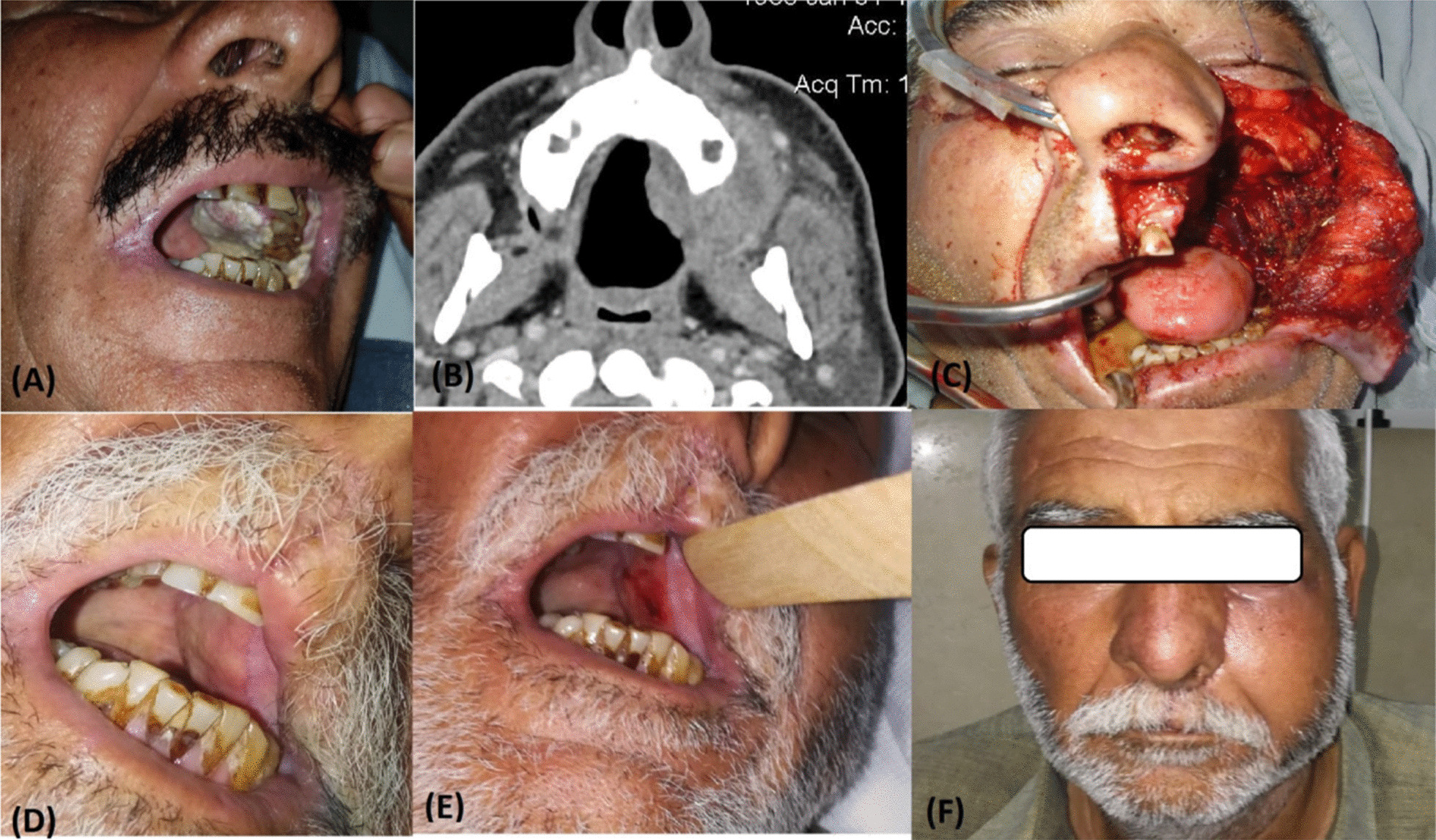
Case 3 **a** Middle age male with large fungating mass of the left maxilla. **b** CT scan showing tumor invading maxillary bone and extending into palate. **c** Intra-operative picture showing large defect after wide local excision. **d** Early followup picture showing temporalis muscle at left palatal half. **e** Picture showing flap mucosalization at 4 weeks follow up. **f** Late follow up picture with good facial contours with mild temporal hollowing

**Fig. 4:**
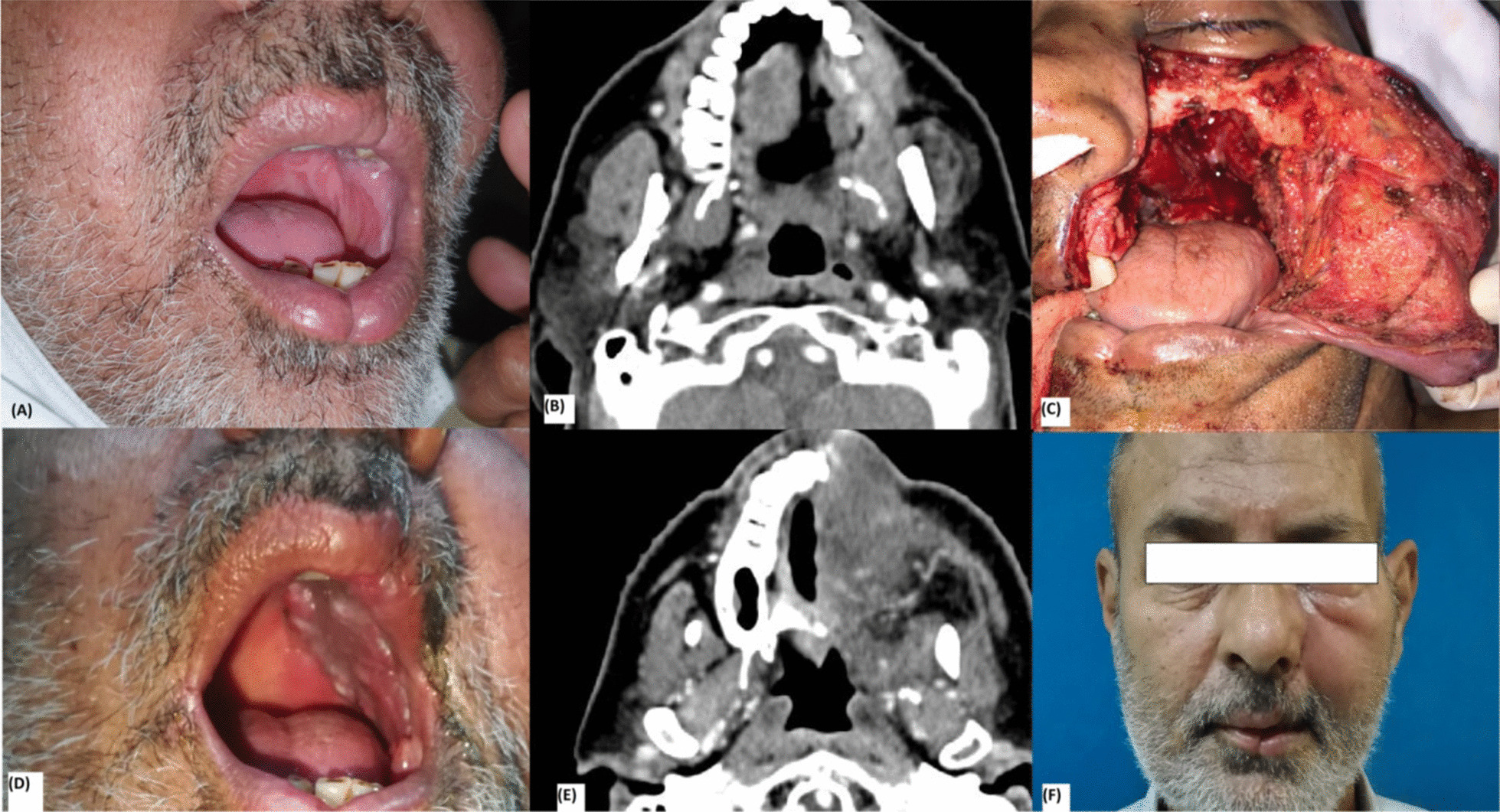
Case 4 **a** Middle aged male with biopsy proven well differentiated squamous cell carcinoma of the left maxilla. **b** CT scan with contrast showing enhancement in the left maxilla sinus, left sided palate with destruction of the left maxillary arch. **c** Picture showing a moderate volume with large surface area defect after wide local excision of the tumor (subtotal maxillectomy) including resection of maxillary arch, palate and anterior and lateral walls with preservation of the orbital floor. **d** Four weeks follow up picture showing good mucosalisation of the temporal muscle flap. **e** Follow up CT scan of the patient showing temporalis muscle flap filling the defect. **f** Late follow up picture, patient has good facial contours with mild temporal hollowing

**Fig. 5:**
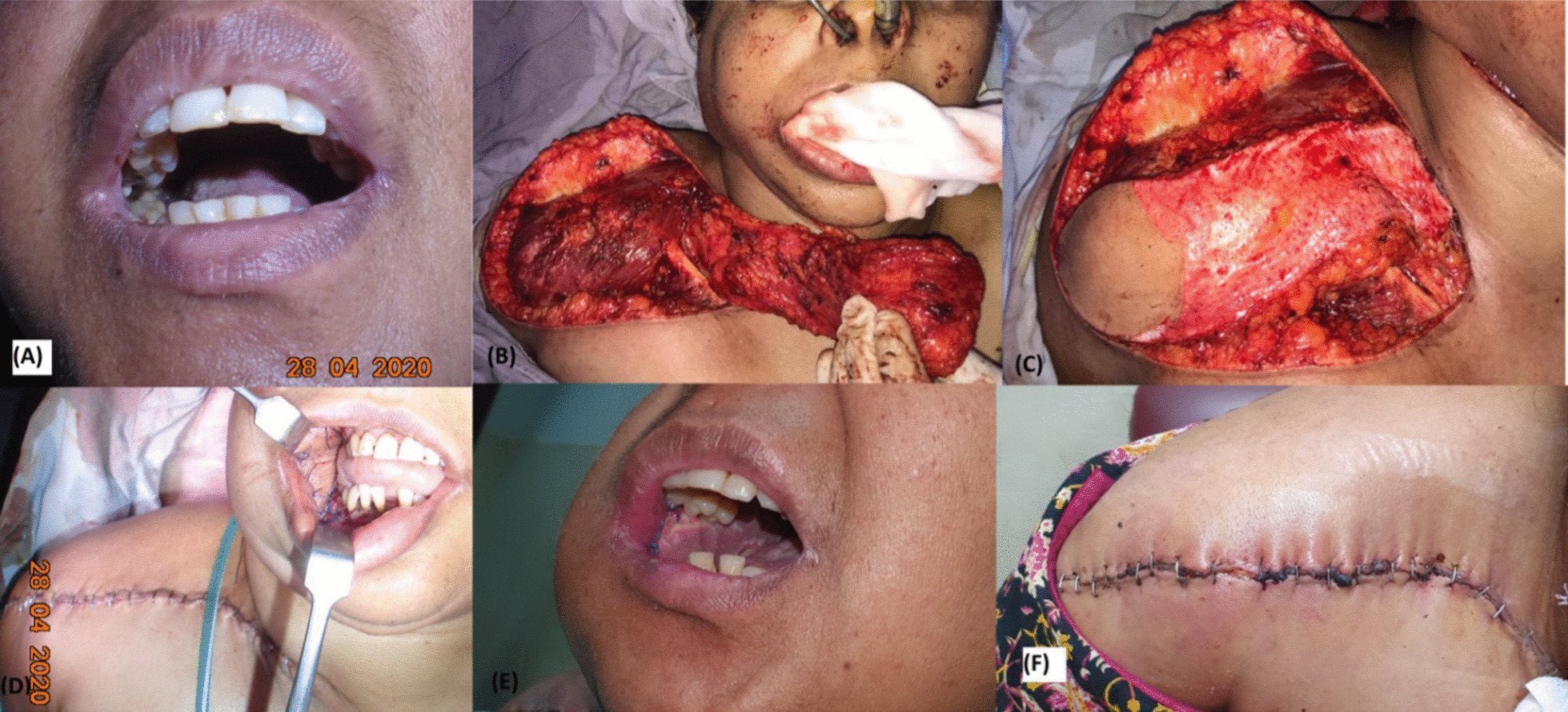
Case 5 **a** A young female patient presented with an ulcerative lesion of the right buccal mucosa, Biopsy reported well differentiated squamous cell carcinoma. **b** After resection of the tumor, An extended supraclavicular flap was elevated. **c** De-epithelialization of the proximal part of the flap was done, which was tunneled under the neck skin into the defect. **d** picture showing the flap inset into the defect and primary closure of the donor site. **e**, **f** 2 weeks follow up of the patient showing right sided cheek edema with good flap mucosalisation and good healing of the donor area

**Fig. 6:**
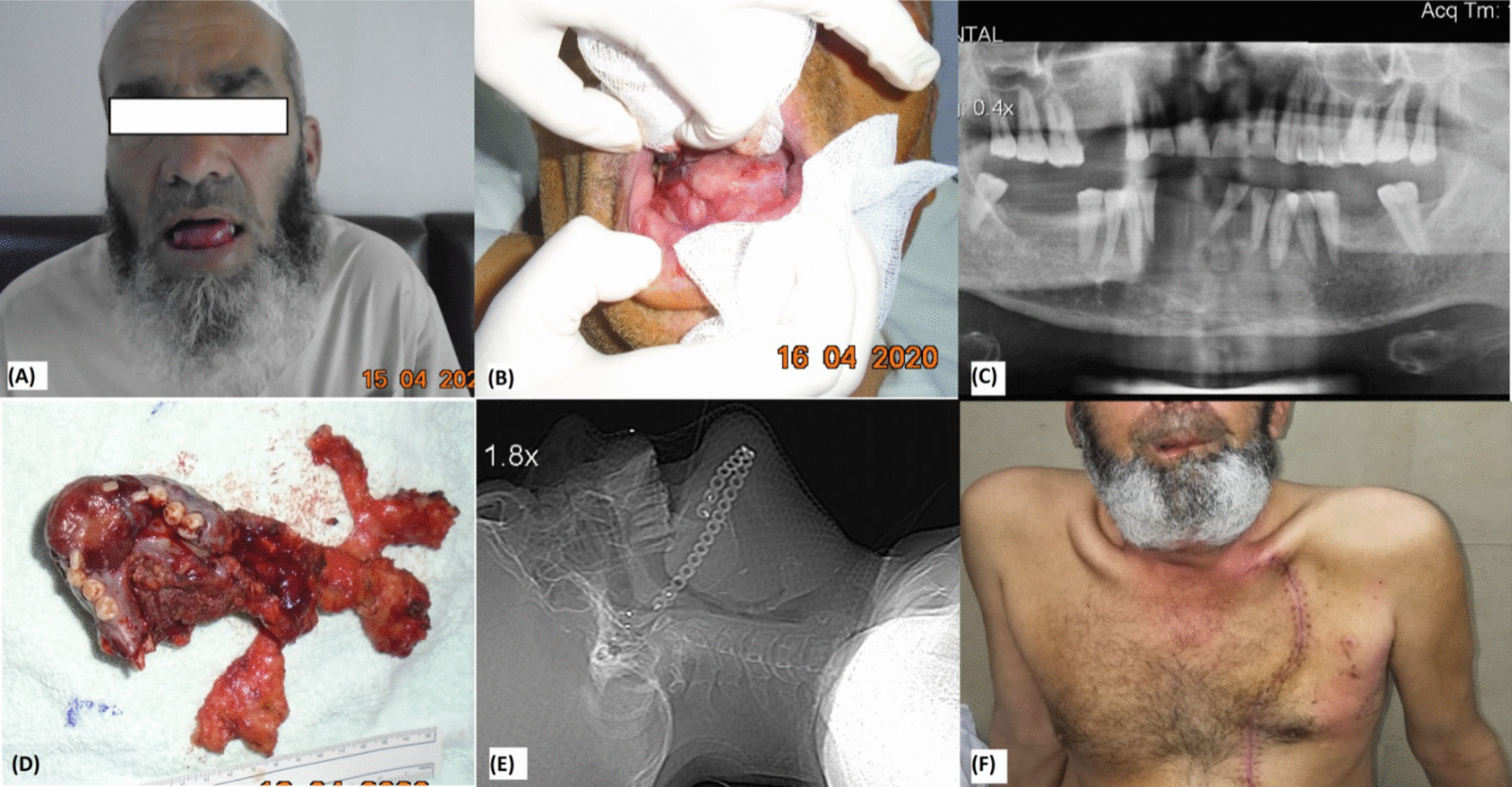
Case 6 **a** Patient with squamous cell carcinoma of body of the right mandible with left mandible extension with involvement of the floor of the mouth (T4a) **b** He had limited mouth opening with restricted tongue movements **c** Orthopantomogram (OPG) showing cortical destruction around the mandibular symphysis. **d** Enbloc Resected specimen showing mandibular arch with part of the floor of the mouth and bilateral neck nodes. **e** right lateral Xray view showing the reconstructed lower jaw with metallic reconstruction plate. **f** soft tissue coverage was provided with myocutaneous pectoralis major flap. Picture showing healed donor site

**Fig. 7:**
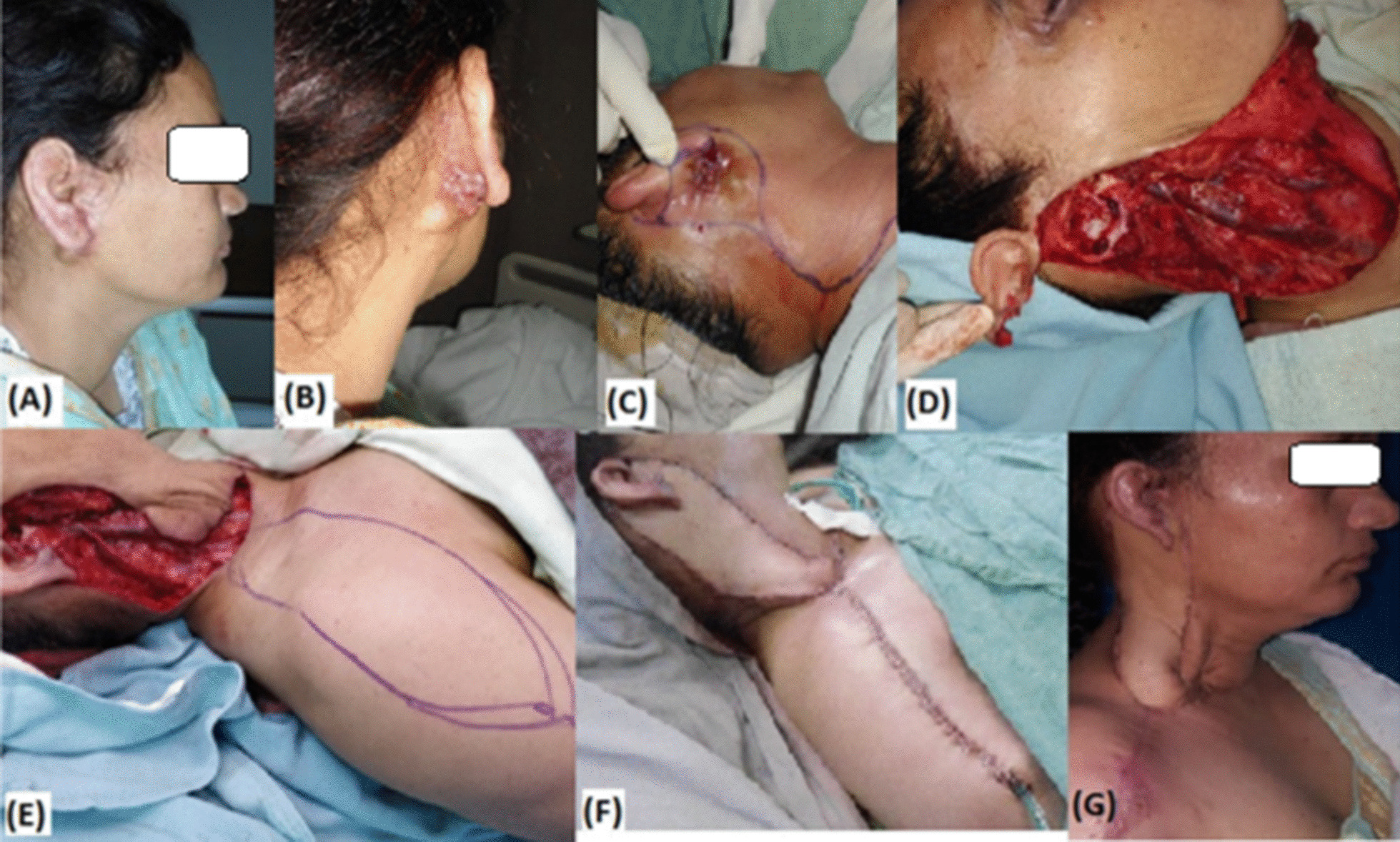
Case 7 **a** A young female patient presented with diagnosed case of adenoid cystic carcinoma of the right parotid with involvement of the middle ear **b** lateral view showing the extension of the tumor into the mastoid area. **c** Marking of the resection with 1 cm margins. **d** Excision involved peri-auricular skin, superficial parotidectomy, external and internal auditory meatus. Ear was intact by a bridge of skin at the root of the helix. **e** A supraclavicular artery flap was designed according to the defect size **f** Picture showing flap inset and donor site closure over a Redivac drain **g** Lateral view of patient at 1 month follow up

**Fig. 8:**
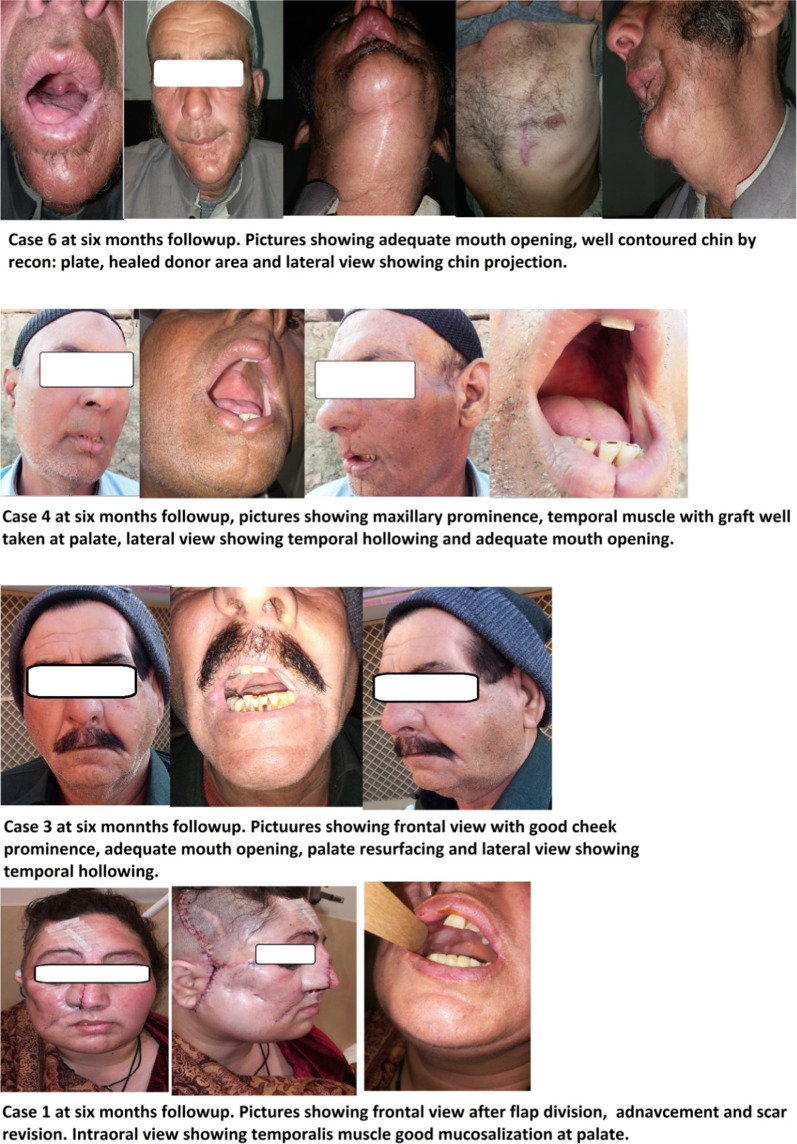
Shows follow-up pictures of same patients at 6 months

## Discussion

The world has witnessed many global health challenges since the beginning of time. The COVID-19 also called as ‘’Severe acute respiratory syndrome 2 (SARS-2)’’ is one of special kinds that has some unique characteristics [8]. It is highly contagious, sprightly transmittable and has inherent stealth properties. The common mode of transmission is by direct human to human contact, aerosol generation, respiratory droplets, oro-fecal route and contaminated surfaces in our environment [9–15]. Reverse transcription Polymerase chain reaction (rRT-PCR) nasal swab tests’ is usually done to detect infection [16].

Recent studies show that rRT-PCR has a sensitivity of 56–83%. This means that 20–40% of the actually infected people will not be detected by this test [17, 18]. This could lead to a false sense of security among health care workers. Asymptomatic, but viral shedding of the initial stage of the infection is usually not detected with routine tests.

Due to the covert and highly contagious nature of this virus, the screening and containment strategies are not well-effective [19].

The infection can be transmitted from a person who is totally asymptomatic [19, 20]. Many studies show that Corona virus is present in body fluids and blood. [10, 11, 21–24] Consequently, all procedures involving bone cutting/ drilling machines, unipolar and bipolar thermal cautery machines, especially in head and neck region have a high risk of infectious aerosol generation [24, 25].

Hence, any surgeries on such patients put the Health care workers (HCW) at high risk of contracting the infection. About 4% of the affected population in China was HCW, and this number was even higher in Italy i.e., 9%. [26, 27] More than 500 health care workers contracted COVID-19 by the start of May 2020 in Pakistan [28]. In these prevailing situation hospitals worldwide are redefining guidelines for elective surgeries.

In the United States of America all the elective cases were cancelled, following recent guidelines published by the American Society of Plastic Surgeons, American Medical Association and American College of Surgeons [22, 29, 30].

Similarly, in Pakistan all the major centers postponed their elective surgical cases and outdoor clinics were closed for the first 6 weeks. Only emergency cases were accepted in the vast majority of hospitals. After 2 weeks, telemedicine outdoor units started at our center to reduce un-necessary contact with patients. Virtual video link consultations were open for patients suspected of malignancies.

Delaying treatment of patients with Head and Neck cancers has a significant impact on the outcome and prognosis, such as Squamous Cell Carcinoma (SCC) of the Head and neck, which is time critical and top priority [31].

The tumor doubling time in head and neck cancer is 1 to 3 months; so many patients who would be operable now would not be operable after 3–4 months. [32, 33] However patients undergoing lengthy surgery, including head and neck surgery, are at high risk of contracting COVID-19 pneumonia and may lead to severe respiratory disease and even death [34–36].

British Association of head and neck Oncologists (BAHNO) guidelines for Head and Neck Surgeries published in March 2020 provide valuable points like, restricting procedures which require post-op HDU/ ITU, reducing duration of surgeries where possible, using local/pedicled flaps instead of free flaps, restricting the number of staff in the operating room and ensuring PPE worn by all staff [37].

Worldwide, many centers have adapted different protocols for using Personal protective equipment (PPE) for surgeries [38]. In order to minimize the spread of infection during surgery, all HCW (Surgeon, assisting residents, assisting nurses and anesthetist) involved in these surgeries used particulate filtering masks (e.g. N95), eyes protective goggles/face shields, disposable surgical gowns. At the same time, in order to minimize surgery duration the surgeon and the assisting team were not allowed to take breaks, once scrubbed in. The operation rooms were transformed into negative pressure rooms. Where possible, patients’ oro-nasal cavities were packed and sealed with adhesive dressings. The use of thermal cautery and powered saw/drill was limited. Tracheostomy was avoided where possible. Table 3 enlists goals of our approach in COVID-19 pandemic.

**Table 3:** Goals of our approach

1	Reduce use of PPE
2	Minimize anesthesia duration and hospital length of stay
3	Less usage of ICU resources
4	Decrease resident/ nurse exposure with frequent flap checks
5	Minimize potential for micro-vascular related take backs

After discussing with the ethical committee, tumor board members and anesthetists, we had to set up our own guidelines and criteria, before operating on these patients. Table 4 is the exclusion criteria for surgery. Patients who fulfilled the criteria were operated upon immediately and the remaining were considered for adjuvant therapies. Preoperatively real-time reverse transcription polymerase chain reaction (rRT-PCR) nasal swabs were taken and high resolution CT scan (HRCT) was done to screen for COVID-19 status. Patients in which these investigations were not done, like in emergency and life threatening situations, were considered COVID-19 positive.

**Table 4:** Following criterion should be considered as exclusion for surgery

1	Age	More than 65 years
2	Co-morbidities/anesthesia tolerance	More than two/high risk
3	Prognosis	Less than 60% survival over next 2 years
4	Site/ neck status	Hypophayrnygeal tumors or neck N3 disease
5	Defect size	Large defect mandating free flap
6	COVID-19 status	HRCT with ground glass opacities or rRT-PCR positive

All These factors were taken into consideration before interacting and planning treatment in these patients. We received about 31 patients in 3 months, who presented with different head and neck pathologies. A focused and thorough clinical examination was done in all patients with the examining doctor in full PPE. All these patients were evaluated based on age, co-morbidities, anesthesia tolerance, disease extent and prognosis.

The common reconstructive options for head and neck reconstruction include microvascular free tissue transfer, regional flaps and occasionally local flaps.

Currently micro-vascular free tissue transfer is considered standard of care and has an advantage of better functional and aesthetic outcomes with less donor-site morbidity [39]. In the current situation, free flaps have the disadvantages of more complex reconstruction with higher chances of aerosol generation, two team approaches with staff overcrowding and time consuming. Therefore, free laps are less suitable in the current pandemic situation. On the other hand pedicled flaps are well established and time honored. They fell into disuse with the advent of microvascular free flaps. The pedicled flaps are reliable and comparable in many aspects with the free flaps for the reconstruction of head and neck defects [40, 41].

Keeping patient and HCW safety as the top priority, the operative protocols were modified. The surgical resection part of the procedure remains standard. As getting a clear margin is always a priority and cannot be altered to reduce time. Reconstruction however, can be tailored to meet the challenges with current constraints. This reduces inter-personnel exposure time, less complicated reconstruction with local or regional pedicled flaps (thus avoiding anastomosis and take backs in case there is flap ischemia) instead of free flaps. Local flaps require less frequent monitoring of flaps and early discharge. These factors not only benefit HCW and patients but also hospital resources.

Also there are certain problems which are specific to plastic surgeons in this COVID-19 pandemic. PPE can physically interfere with the surgeon’s loupes and head lights. PPE can be very uncomfortable in lengthy head and neck reconstruction cases. And bifocal microscopes/ loupes cannot be used simultaneously with face shields. This certainly affects the choice of reconstruction.

The Head and neck surgeon with one assistant did the resection part in all cases. Tracheostomy was done in 17 (54.8%) patients. The mean time for the resection was 207 min. Each resection was done under frozen control. Without any breaks, the reconstruction started in all cases. Loco-regional pedicled flaps (Pectoralis major muscle flap, supraclavicular flap, temporalis muscle flap, naso-labial flap and forehead flap) were used for reconstruction of different defects (maxillectomy, glossectomy, buccal mucosa, floor of mouth). To reduce time of surgery and inter-personnel exposure, metal reconstruction plates were used in three patients.

Mahieu et al. in their study compared the outcomes of pedicled flaps with free flaps, for head and neck reconstruction. According to them, the functional outcomes and complication rates were comparable [40]. Even in some circumstances local pedicled flaps are preferred over free flaps, such as patients who cannot tolerate lengthy general anesthesia and with co-morbids [42]. In many circumstances regional flaps prove to be reliable and less expensive as compared to the free flaps [41].

Pectoralis major muscle flap has adequate bulk, and it easily adheres to irregular 3 dimensional defects. It can be used to wrap around metal recon plate to avoid plate extrusion and reconstruct the floor of the mouth. We have used it for coverage of chin defect, reconstructing the floor of mouth and enfolding the reconstruction plates. It provided as filling dead space and reliable soft tissue coverage.

For reconstruction of palate, buccal mucosa, retro-molar trigone and mastoidectomy defects, we have used Temporalis muscle flap, which provided adequate surface coverage and mucosalization was seen after 4 weeks in patients. The temporalis muscle flap is an all-round flap for craniofacial defects like orbit, the lateral base of the skull and oral cavity. This flap is advantageous because of the close vicinity and robust blood supply. Temporalis muscle flap is preferred in situations where free tissue transfer cannot be done [43].

Temporalis muscle flap has less donor site morbidity with hidden scar and no obvious functional deformity [44]. Tara Brennan et al. and Jesse E et al. showed very good results of temporalis muscle flap for reconstruction of palatal and tongue base defects. It has a high success rate and an outstanding alternative in the reconstruction of difficult intra-oral defects [45, 46]. In cases where a hair-free and less bulky flap is needed, temporalis muscle flap has superior results over the pectoralis major muscle flap [47].

In our patients where large buccal mucosa, cheek and floor of mouth defects were created, a supraclavicular artery flap was used. Although, it has some limitations in length, we didn’t experience any problem in distal flap circulation. It was first described by Lamberty in 1979 [48].

The supraclavicular artery flap provides large thin fascio-cutaneous coverage to most of the defects of the head and neck region [49]. This flap is easy to harvest and a good colour match if used for skin defects [50]. Super-charge of flap can be done if a large size flap is needed [51].

A combination of different flaps was used in one patient who presented to us with a large composite defect of cheek, maxilla, and nasal wall. She is a known case of Mucormyscosis, treated with multiple debridements. A forehead flap was used to reconstruct side of nose, cheek rotation flap used to cover cheek defect and temporalis muscle flap was used to reconstruct palate. She recovered well from the surgery. The temporalis flap had good mucosalization in fourth week.

Whenever there is need, always utilize a combination of flaps technique, which gives similar type of tissue readily available in the vicinity and scars / patches, can be hidden in facial aesthetic units.

In order to reduce duration of surgery, metal plate reconstruction of mandible is a favorable option. Three of our patients had mandibular (hemi-mandible n = 2, segmental n = 1, marginal n = 1) defects, in which metal reconstruction plates were used. These plates were enfolded with Pectoralis major muscle flaps to avoid extrusion. Saunders et al. in their study of 27 patients presented their success rates of 78–85% with metal reconstruction plate use. [52] Once this pandemic is over, in these patients at a later stage, vascularized free fibula reconstruction can be done.

Regarding chemo-radiotherapy treatment in such patients, critical specialized units should be designated with proper screening counters [53]. These patients are at higher risk of contracting the disease as they are immunocompromised, where possible telemedicine services should be utilized [54].

In disaster scenarios or health crises that we are facing today in 2020, we are forced to consider, a step down on the reconstructive ladder. The basic essence of our specialty is versatility and adaptability. There is no single best solution to any problem, but the circumstances define what is best at that moment in that particular case. The rapidly ongoing research and observation from across the world should be shared to enlighten the guidelines and experiences about the novel Corona Virus management and prevent complications.

The "resurrection" of local and regional flaps can prove to be a useful adjunct in the reconstruction of composite head and neck defects in current situation.

In this time of historical crisis we should be adherent to the ethical principles of utilitarianism, egalitarianism, fidelity, veracity and respect for people [55].

## Conclusion

Pedicled flaps are proving as the workhorse for head and neck reconstruction in unique global health crisis. Vigilant use of proper PPE and adherence to the ethical principles proves to be the only shield that will benefit patients, HCW and health system. Routine methods must be mastered, but never let them master you. (The Principles and Art of Plastic Surgery).

## Data Availability

All data generated or analyzed during this study are included in this published article. Further information if needed can be requested from corresponding author.
